# *De novo* variants of *IRF2BPL* result in developmental epileptic disorder

**DOI:** 10.1186/s13023-024-03130-z

**Published:** 2024-03-13

**Authors:** Yong Wang, Zhongling Ke, Yufen Li, Mingqi Qiu, Jing Liu, Zuozhen Yang, Shu Wen, Mengmeng Liang, Shan Chen

**Affiliations:** 1 Department of Pediatrics, Fujian Medical University Union Hospital, No. 29, Xinquan Road, Gulou District, 350001 Fuzhou, Fujian China; 2 Department of Pediatrics, Linyi People’s Hospital, 276003 Linyi, Shandong China; 3 Cipher Gene LLC, 100089 Beijing, China

**Keywords:** *IRF2BPL*, Neurodevelopmental disorder, Epilepsy, Zebrafish, Variant

## Abstract

**Background:**

Pathogenic variants of the *IRF2BPL* gene have been reported to cause neurodevelopmental disorders; however, studies focused on *IRF2BPL* in zebrafish are limited.

**Results:**

We reported three probands diagnosed with developmental delay and epilepsy and investigated the role of *IRF2BPL* in neurodevelopmental disorders in zebrafish. The clinical and genetic characteristics of three patients with neurodevelopmental disorder with regression, abnormal movements, loss of speech and seizures (NEDAMSS) were collected. Three *de novo* variants (NM_024496.4: c.1171 C > T, p.Arg391Cys; c.1157 C > T, p.Thr386Met; and c.273_307del, p.Ala92Thrfs*29) were detected and classified as pathogenic or likely pathogenic according to ACMG guidelines. Zebrafish crispants with disruption of the ortholog gene *irf2bpl* demonstrated a reduced body length and spontaneous ictal-like and interictal-like discharges in an electrophysiology study. After their spasms were controlled, they gain some development improvements.

**Conclusion:**

We contribute two new pathogenic variants for *IRF2BPL* related developmental epileptic disorder which provided evidences for genetic counseling. In zebrafish model, we for the first time confirm that disruption of irf2bpl could introduce spontaneous electrographic seizures which mimics key phenotypes in human patients. Our follow-up results suggest that timely cessation of spasmodic seizures can improve the patient’s neurodevelopment.

**Supplementary Information:**

The online version contains supplementary material available at 10.1186/s13023-024-03130-z.

## Introduction

Interferon regulatory factor 2 binding protein-like (IRF2BPL) is a transcription factor that was first identified as C14orf4 and is a 796 amino acid protein with a mass of 82.7 kDa [1]. *IRF2BPL* involvement has been widely reported in neurodevelopmental disorders, including adult-onset dystonia syndromes [2–6] and early-onset child neurodevelopment regression and epilepsy [7, 8]. Patients with *IRF2BPL* gene variants present with regression, abnormal movements, loss of speech, and seizures. Further, loss of function of IRF2BPL results in neural dysfunction, which has been validated in Drosophila and zebrafish [9].

Here, we report three patients with neurodevelopmental delay driven by variants of *IRF2BPL*. In addition, we investigated the contribution of *IRF2BPL* to the phenotype via a zebrafish model.

## Materials and methods

### Patients

This study was approved by the ethics committee boards of Fujian Medical University Union Hospital. Informed consent was obtained from the parents of the patients. All clinical results, including clinical manifestations, electroencephalography (EEG), magnetic resonance imaging (MRI), developmental assessment, routine hospital examinations and genetics testing, were collected and analyzed.

### Whole-exome sequencing and variant analysis

Genomic DNA was extracted from peripheral blood and then fragmented into 200–400 bp fragments. After purification and capture by an IDT exome capture kit, the libraries were sequenced via an Illumina platform. After removing low-quality reads, clean data were obtained and mapped to the human reference genome (GRCh38/hg38) by BWA, and variants were analyzed by GATK and annotated by InterVar (http://wintervar.wglab.org/). All variants with a minor allele frequency of ≤ 0.05 in a public database (ExAC, gnomAD, 1000 Genomes) were selected for pathogenicity evaluation according to ACMG guidelines. Sanger sequencing was performed to validate the candidate variants in the probands and their families.

### Zebrafish maintenance

Adult zebrafish were maintained in tanks with circulating water at 28 °C and a light/dark cycle of 14/10 hours, and all fishes were fed twice per day. Zebrafish embryos were harvested by mating adult fish following standard protocols [10]. Larvae were cultured in embryo media with 0.03% Instant Ocean and 0.0002% methylene blue. Fluorescent imaging experiments were performed on the Tg(HuC:eGFP) line [11]. All experimental procedures were performed according to the Guide for the Care and Use of Animals (th 2011).

### Gene editing and TIDE assessment

The orthologous gene of *IRF2BPL* in the zebrafish genome was identified as *irf2bpl* (ENSDARG00000004297) by the DIOPT Ortholog Finder (https://www.flyrnai.org/cgi-bin/DRSC_orthologs.pl), and the protein identity between human *IRF2BPL* and the zebrafish ortholog was 66%. Then, single guide RNA (sgRNA) targets were identified using the CHOPCHOP (version 3) online tool (https://chopchop.cbu.uib.no) [12] and ordered from GenScript (Nanjing, China). Three sgRNAs were designed for the targeted gene as follows (PAM sequence in lowercase letters): CGGCGCAGGTCTCCTCGTCGagg, CAGGGGTTGCGTGAATTACGagg, and AGGGGTTGCGTGAATTACGAggg. To generate mutagenesis in the targeted gene, fertilized embryos (1–2 cell stage) were injected with ~ 2 nL CRISPR complexes composed of three sgRNAs (100 ng/µL of each sgRNA) and Cas9 protein (250 ng/µL). Twenty-four hours after injection, a few embryos from the injected group were pooled and sequenced by Sanger sequencing to verify the mutagenesis efficacy via the TIDE (Tracking of Indels by DEcomposition) online tool (https://tide.nki.nl) [13]. Individual larvae were collected for sequencing and TIDE analysis to confirm mutagenesis. Larvae with TIDE efficacy < 5% were excluded from the subsequent phenotypic data analysis.

### Imaging

At 5 days post-fertilization (dpf), Tg (HuC: eGFP) zebrafish larvae were placed in a customized mini-well plate (5 mm diameter; 1 mm depth, 1 fish/well) dorsal side up for bright-field and fluorescent imaging. Images were obtained with a Touptek CCD camera and Nikon SMZ800N stereo fluorescence microscope with 2X magnification for bright-field imaging and 4X magnification for fluorescent imaging. Images were then analyzed in Fiji (ImageJ) to determine eye distance, body length and central nervous system (CNS) area measurements.

### Electrophysiology

Zebrafish larvae at 5–6 dpf were paralyzed in 300 µM pancuronium (Sigma), immobilized in 2% low-melting-point agarose in a recording chamber, filled with embryo media and transferred to an electrophysiology platform. Local field potential (LFP) data were obtained from the optic tectum using a glass microelectrode. Electrodes were filled with 2 M NaCl, and electrical activity was recorded using an extracellular amplifier with a high impedance head stage (1700, A-M Systems). Signals were low-pass filtered at 5 kHz, high-pass filtered at 1 Hz, and digitized at 10 kHz using a digital acquisition board (Measurement Computing). Data were recorded and analyzed with DClamp (https://sites.google.com/site/dclampsoftware/home). As described in a previous study [14], ictal-like events were defined as electrical events greater than 5x baseline noise, multispike and > 500 ms in duration. Interictal-like events were defined as smaller discharges than ictal-like events with amplitudes greater than 3x baseline noise and > 100 ms in duration. Occurrences of electrographic epileptiform events (ictal-like events + interictal-like events) were analyzed.

### Statistics

Statistical analysis was performed in Prism 8 (GraphPad Software). The unpaired T test was used for comparing two variable groups. The chi-square test was used for epileptiform event incidence analysis. Significance for all tests was defined as *p < 0.05; **p < 0.01; ***p < 0.001.

## Results

### Clinical features

**Fig. 1:**
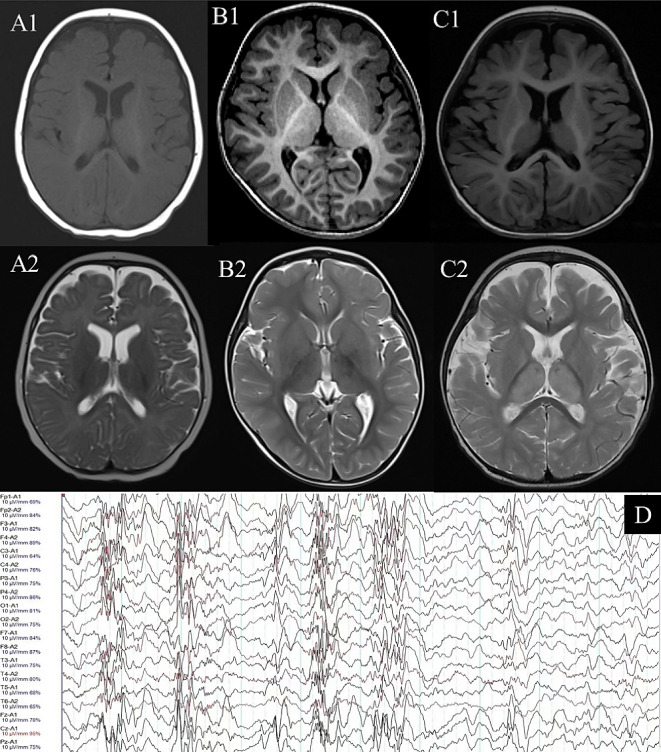
Clinical features of three probands. **A1**, **A2**: MRIs for case 1 at 8months. MRI revealed widened subdural space of bilateral frontal and temporal, and slightly expanded ventricular system. **B1**, **B2**: Normal MRI for case 2 at 2 years and 2 months. MRI revealed slightly wider at subarachnoid space of the temporal pole. **C1**, **C2**: MRI for case 3 at 1 years and 1months. MRI revealed slightly wider at subarachnoid space of the frontal and temporal pole. **D**: Typical hypsarrhythmia EEG

The age at seizure onset of the patients ranged from five to nineteen months. Two probands had onset before 8 months, indicating early-onset epilepsy. All patients exhibited epilepsy, intellectual disability (ID), developmental delay (DD), language disability and developmental regression, and two patients exhibited movement abnormalities. MRI revealed brain structural abnormalities in two probands. The clinical details are summarized in Fig. 1; Table 1.

**Table 1:** Clinical features of patients with *IRF2BPL* variants

Clinical features	Patient 1	Patient 2	Patient 3
Variant	IRF2BPL: NM_024496.4, c.1171 C > T p.Arg391Cys	IRF2BPL: NM_024496.4, c.273_307del p.Ala92Thrfs*29	IRF2BPL: NM_024496.4, c.1157 C > T p.Thr386Met
Gender	Male	Male	Male
ACMG classification	Likely pathogenic	Pathogenic	Likely pathogenic
Current age	14month	2 year-6-month	13 months
Growth for age at most recent visit	Weighted 9.3 kg, hight 73 cm at 14 months	Weighted 12 kg hight 88 cm at 2 year-6-month	Weighted 10 kg hight 79 cm at 13 months;
Development delays preceding regression	Yes	Yes	Yes
Age of onset of motor regression	5 month	1 year and 5 month	8 month
Current speech and language skills	Only speak “baba, mama”	Only speak “Papa Mama”	No language
Current gross motor skills	Start climb	Could not sit alone, climb and grasp	Sit alone and hold his head
Seizures	Spasm combined with focal epilepsy, currently under control	Typical spasm. minor seizures were observed after reduced antiepileptic drugs	Spasm combined with focal epilepsy
Movement abnormalities	No dyskinesia	Dyskinesia	No dyskinesia
Other neurological findings	None	None	Hypertonia
EEG	Abnormal EEG of infants, hypsarrhythmia, frequent series of epileptic spasmss and focal onset seizures were observed	Hypsarrhythmia, several isolated seizures were observed	Hypsarrhythmia. Frequent series of spasm, spasm mixed with wandering spasm were observed
Brain MRI	Bilateral frontal and temporal subdural spaces were widened	Normal	The subarachnoid space of the frontal and temporal pole is slightly wider

#### Case 1

The proband was a five-month-old boy who was referred to our hospital for recurrent convulsions. He was the second child from Chinese parents with a nonconsanguineous and no family history of genetic disease and delivered at full-term via spontaneous delivery by his 31-year-old healthy mother. At the age of 3 months, he could raise his head steadily and follow lights and sounds well. At five months, epileptic spasms were observed before going to sleep or when waking up and appeared as a cluster, with a frequency of 10–34 times per day. After the spasm attack, he experienced developmental regression, manifested as unsteadiness in raising his head, slow reaction to human faces, and inability to find the source of sounds.

Physical examination at 5 months revealed a normal weight (8 kg, ref: 6.5 ~ 9.9 kg), height (67 cm, ref: 62.3 ~ 72.6 cm) and head circumference (42 cm, ref: 40.2 ~ 45.0 cm). Electroencephalography (EEG) documented hypsarrhythmia. Neuropsychological development (Gesell Developmental Schedules) and the development quotient (DQ) assessment revealed development delay: total, 51.7; gross motor, 44.6; fine motor, 53.5; adaptability, 35.7; language ability, 71.4; and social ability, 53.5.

At the age of eight months, brain MRI revealed a widened subdural space of the bilateral frontal and temporal lobes and a slightly expanded ventricular system.

Finally, the child was diagnosed with infantile epileptic spasms syndrome (IESS) and treated with ACTH hormones and vigabatrin successively, but the seizures could not be controlled. After treatment with methylprednisolone (20 mg gradually increased to 40 mg (5 mg/kg/d)) for 4 weeks and vitamin B6 (100 mg qd) for 7 days, the seizures and atypical hypsarrhythmia remained. Further vigabatrin treatment (1250 mg qd) was added, and the seizure symptoms disappeared within 2 weeks.

After spasms disappeared, the neurodevelopment gradually improved. When follow-up at 14 months, the child was treated with vigabatrin for maintenance and remained seizure-free. Although development was still lagging, the child could crawl, stand, and pronounce the sounds of “baba” and “mama”.

Now he is 3 years old, he gains gradual progress in development and acquisition of motor function and some language abilities, although his development still delayed around 10 months comparing with other children. The Gesell scale (36 months) indicated developmental age (DA), adaptability, 23.2 months; major exercise, 23.6 months; fine motor, 29.6 months; language, 25 months; and personal social, 25.7 months.

#### Case 2

The proband was a four-year-old boy. He suffered recurrent convulsions for 2 months when he was admitted to the hospital (1 year and 7 months). His epileptic spasms exhibited as a cluster, with a frequency of 14 times per day. Electroencephalography (EEG) documented hypsarrhythmia.

The proband exhibited hypotonia in the neonatal stage and was suspected to have floppy infant syndrome. His motor development was delayed. Although rehabilitation training was actively carried out, the progress was slow. He was able to hold his head and sit only when leaning against something. When he was 1 year and 5 months old, he exhibited epileptic spasms, motor function regression, and an inability to hold his head up, sit or roll over.

After admission, he was diagnosed with infantile epileptic spasms syndrome (IESS) and treated with a ketogenic diet, ACTH and antiseizure medications, including levetiracetam, oxcarbazepine, vigabatrin, clobazam, clonazepam and pirampanide successively. After being controlled by ACTH for three days and vigabatrin (100 mg/kg/d) for two weeks, nodding convulsions recurred. At the age of 2 years and 2 months, brain MRI was performed, and the results were normal. At the age of 2 years and 6 months, corpus callosotomy was performed to eliminate the seizures. The epileptic spasms were effectively controlled; however, intellectual development was still delayed. His neurodevelopment has improved, gradually acquired motor functions such as standing and walking, acquired the ability to repeat words, understand a small amount of daily language, and communication with his family.

#### Case 3

The proband was a ten-month-old boy who was referred to the hospital because of recurrent convulsions for two months. He was the first child of nonconsanguineous Chinese parents, delivered by cesarean section because of abnormal fetal position. Newborn physical examination revealed serious abnormalities, including congenital bilateral hip dislocation, jaundice of the newborn, patent ductus arteriosus, patent foramen ovale and micrognathia. EEG showed hypsarrhythmia, and a series of epileptic spasms and several focal onset seizures were detected.

Before onset, the proband was able to listen, look, eat, laugh, hold his head up and roll over when he was 7–8 months old. Approximately 2–4 weeks after onset, he gradually lost the ability to follow sound or light and to laugh. Intellectual development began to show regression after the onset of spasms.

Finally, he was diagnosed with developmental epileptic encephalopathy and infantile spasm. After a 21-day course of ACTH (25 IU qd) treatment, the spasms were relieved but did not disappear. Therefore, topiramate (25 mg bid) was administered. At 13 months old, the patient still had one spasm attack per day, but his mental state and development improved. He gained the ability to sit when leaning against something but failed in language development. Brain MRI revealed a slightly wider subarachnoid space of the frontal and temporal poles.

### Identification of IRF2BPL variants

We performed WES and Sanger sequencing to identify rare variants that could explain the infantile spasms in our patients. The patients in our study were found to carry *de novo* variants in IRF2BPL by trio WES (Table 1). Two variants were missense variants (NM_024496.4: c.1171 C > T, p.Arg391Cys; NM_024496.4: c.1157 C > T, p.Thr386Met), and one variant was a frameshift variant (NM_024496.4: c.273_307del, p.Ala92Thrfs*29). The variants c.1171 C > T and c.1157 C > T were classified as likely pathogenic (LP) because they were *de novo* variants (PS2), had extremely low frequency in public databases (PM2_supporting) and were predicted as damaging by a multiple prediction algorithm (PP3). The c.273_307del variant was classified as pathogenic (P) because it was a *de novo* variant (PS2), was a frameshift variant that resulted in early translation termination (PVS1_Strong) and had an extremely low frequency in public databases (PM2_supporting). All these variants were *de novo* and were verified by Sanger sequencing.

### Zebrafish validation

**Fig. 2:**
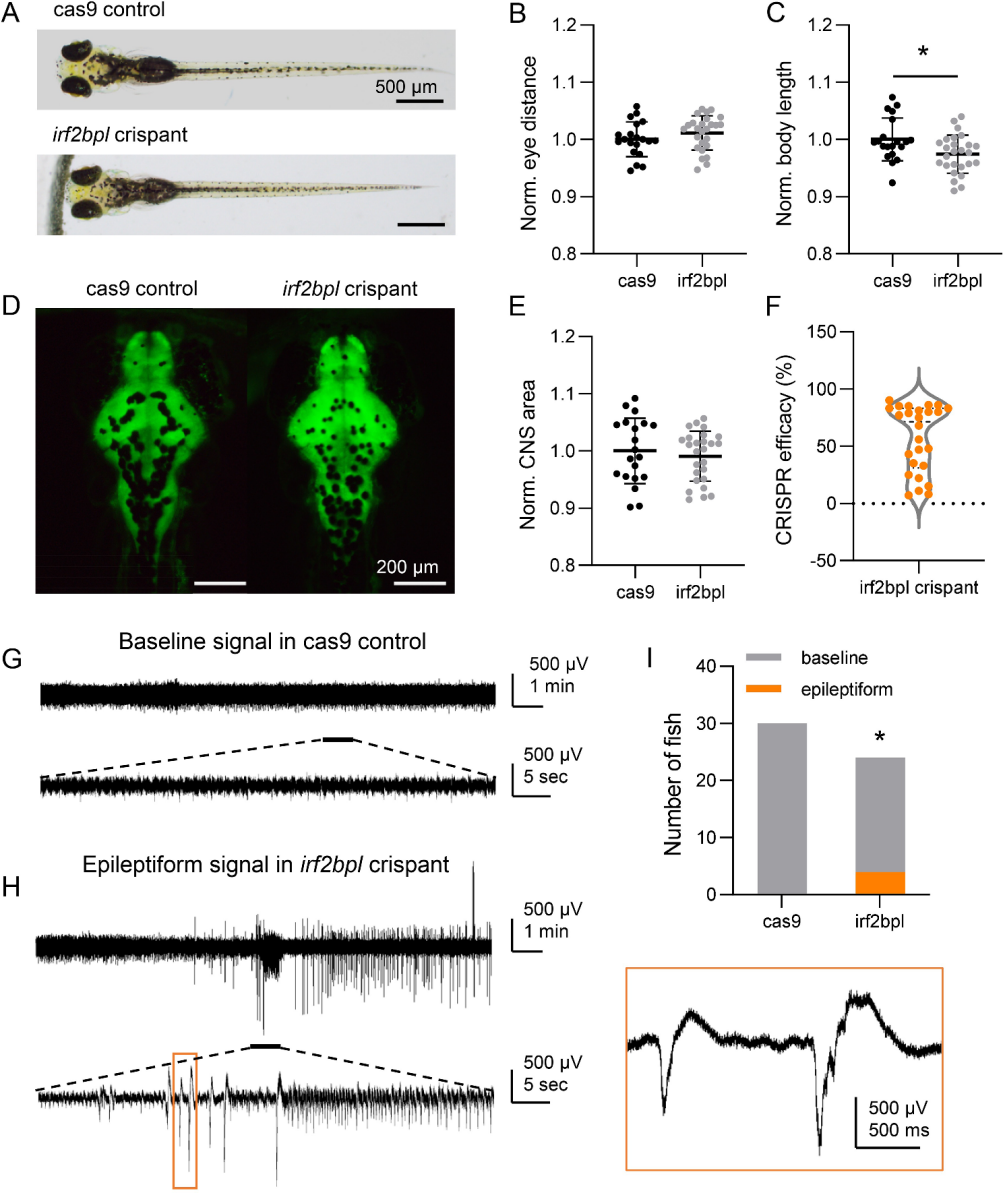
Disruption of Zebrafish irf2bpl Leads to Spontaneous Seizures. **A**. Representative bright-field imaging of larval zebrafish at 5 days post fertilization (dpf). Top, cas9 injected control; bottom, irf2bpl F0 CRISPR (crispant). **B**-**C**. Measurements of eye distance and body length in cas9 injected control (*n* = 20 fish) vs. irf2bpl crispants (*n* = 26 fish; unpaired t test). Data were normalized to the average values of cas9 control group. **D**. Representative imaging of HuC: eGFP expressed larval zebrafish shows CNS fluorescence pattern at 5 dpf (dorsal view). Left, cas9 injected control; right, irf2bpl crispant. **E**. Normalized CNS area in cas9 injected controls vs. irf2bpl crispants. Data were normalized to the average CNS area of cas9 control group. **F**. CRISPR efficacy calculated via TIDE method of individual irf2bpl crispant used in phenotypic study (n = 26 fish). **G**. Representative local field potential (LFP) recording from cas9 injected control shows baseline activity. **H**. Representative LFP recording from irf2bpl crispants shows epileptiform events, and a magnified view of the orange box is shown. **I**. Occurrence of electrographic epileptiform events in cas9 injected control (n = 30 fish) and irf2bpl crispants (n = 24 fish; Chi-square test). Grey, number of fish showed baseline activity; Orange, number of fish showed epileptiform activity. Scale bars as indicated in the figure. Error bars indicate standard deviation (SD). Statistical significance is indicated as *p < 0.05, **p < 0.01, ***p < 0.001

We investigated the function of irf2bpl, the ortholog of human IRF2BPL, in developing zebrafish larvae. We performed CRISPR‒Cas9 genome editing and characterized the morphological and neurological phenotypes in zebrafish F0 CRISPR (crispant) at 5–6 days post-fertilization (dpf; Fig. 2). Compared with the Cas9-injected control group, irf2bpl crispants showed no difference in eye distance (Fig. 2B; *n* = 20 and 26 for Cas9 controls and irf2bpl crispants, respectively; unpaired t test, *p* = 0.2202) but exhibited significantly reduced body length (Fig. 2C; unpaired t test, *p* = 0.0169). We further examined CNS morphology, and no significant difference was observed between the Cas9 control and irf2bpl crispant (Fig. 2D; unpaired t test, *p* = 0.5381). In the electrophysiology study, spontaneous ictal-like and interictal-like discharges, i.e., epileptiform events, were observed in irf2bpl crispants (Fig. 2G-I; *n* = 30 and 24 for Cas9 controls and irf2bpl crispants, respectively; chi-square test, *p* = 0.0201). Together, these results indicated the essential role of irf2bpl in zebrafish embryonic development and neurological function.

### Phenotype correlation

A previous study reported that patients with IRF2BPL variants exhibited cerebellar signs (100%), moderate-to-profound ID (88%), and epilepsy (71%). Six (6/34) had epilepsy onset in infancy (median age = 6 months), with spasms followed by focal myoclonic, tonic, and atonic seizures [15]. Our three patients also exhibited early-onset epilepsy (median age = 19 months). In our zebrafish model, irf2bpl-knockdown zebrafish exhibited spontaneous ictal-like and interictal-like discharges during the electrophysiology test, which supported early-onset epilepsy in the previously reported cases and our patients.

## Discussion

The *Irf2bpl* gene encodes a 796 amino acid protein that is widely expressed in many organs, including the central nervous system. Previous studies have demonstrated that *Irf2bpl* functions as a transcriptional activator or E3 ubiquitin ligase [7]. In 2007, Heger et al. found that *Irf2bpl* expression increased in the medial basal hypothalamus of female rhesus monkeys in early puberty and increased further in the middle of puberty [16]. The expression of *irf2bpl* in the hypothalamus was also increased in adolescent female mice. In situ hybridization showed that *irf2bpl* mRNA was abundant in hypothalamic nuclear cells involved in GnRH (152,760) secretion, such as the arcuate nucleus. These results indicated roles of *Irf2bpl* in the central nervous system.

Tran Mau-them et al. [8] discovered 10 *de novo* heterozygous nonsense or frameshift variants in the *Irf2bpl* gene in 11 unrelated patients. RNA analysis of fibroblasts from these patients showed nonsense-mediated RNA degradation, which suggested that short protein fragments were translated.

Marcogliese et al. [7] identified heterozygous variants in the *Irf2bpl* gene in 7 patients with neurodevelopmental disorders, and knockdown in Drosophila resulted in progressive neuromotor and learning dysfunction, which indicated that the *Irf2bpl* gene is involved in neural development and neuronal maintenance. In order to validate the phenotype for loss function of *Irf2bpl* gene, knock-down zebrafish model was constructed. In our zebrafish experiment, we found that the body length of the F0 knockout group was significantly shorter than that of the control group, resulting in a larger eye distance to body length ratio. There was no significant difference in the forebrain, midbrain, and hindbrain areas and the area of the whole brain. The zebrafish did not exhibit malformation in the brain, perhaps because the observation was too early. In our clinical data, all three patients showed developmental regression. Combined with the cases reported by previous researchers, these results confirmed that the *Irf2bpl* gene affects the overall development of patients.

According to the current medical cognitive level, limited evidence has suggested that there is a clear correlation between the genotype of *Irf2bpl* gene variants and the severity of clinical manifestations or drug treatment responsiveness. However, the genotype helps to establish clinical diagnosis and prognosis. Because developmental epileptic encephalopathy widely influences the brain development process in infancy, the possibility of poor prognosis is high. It was necessary to try the available treatment strategies and epilepsy surgical intervention as early as possible after diagnosis to improve the prognosis at the critical window of brain development.

*IRF2BPL* gene defects affect the brain development process in early infancy, leading to overall developmental retardation, while brain function regression is closely related to epileptic spasm and dysrhythmia on EEG. The overall course of this disease was consistent with the manifestation of developmental epileptic encephalopathy. The heterogeneity of the clinical phenotype was related to the mutation burden of brain neuron cells after *IRF2BPL* gene mutation, variant allele frequency, complex incremental migration process, etc. [17].. The most common characteristics in children were growth retardation and intractable epileptic seizures, while in the older age group, MRI showed signs of abnormal brain structure.

In this study, all three patients exhibited developmental and epileptic encephalopathy caused by *IRF2BPL* gene variants, and the severity of the disease varied. Epileptic convulsion with abnormal EEG and general developmental delay are two important phenotypes in infancy that seriously affect the brain development process and lead to resistance to various antiepileptic drugs. Generally, the treatment plan for epileptic spasm should be initiated as early as possible. First-line strategies, including ACTH, VGB and even combination therapy (hormonal treatment with vigabatrin), may be used to evaluate the effectiveness. There might be a certain response rate of vigabatrin in these children, which is crucial for brain development.

After comparison with previous reports, we found that the variation in the *IRF2BPL* gene was significantly associated with developmental regression. In previously reported cases, variation in the *IRF2BPL* gene was associated with major clinical phenotypes such as epilepsy, dystonia, ataxia, spasms and a variety of ocular disturbances [18]. Among these patients, there were six cases with an onset age under 1 year (excluding our cases), and the onset age did not seem to be significantly correlated with the clinical phenotype or severity of the disease. In patients with the epileptic phenotype, the proportion of MRI abnormalities was higher. In a recent report, several cases with progressive myoclonic epilepsy associated with the IRF2BPL gene were found to have onset in adulthood [19]. Therefore, the clinical heterogeneity of IRF2BPL gene variation is high, and more detailed clinical cases are needed to enrich the correlation between genotype and phenotype.

The reported clinical phenotypes were significantly heterogeneous and related to onset and survival age. To date, there has been no effective treatment strategy. Multiple epilepsies, including serious spasms, myoclonia and motor dysfunctions, such as dystonia, ataxia, loss of autonomous motor ability, intellectual disability and other serious phenotypes, have been reported. However, the brain and cerebellum atrophy and other abnormalities reported in Marcogliese’s magnetic resonance imaging (MRI) of older patients may be related to the age of the patients [7]. When the children were still in infancy, the first manifestation was most often progressive neurodevelopmental degeneration [20].

Children with *IRF2BPL* variants exhibit highly heterogeneity, complex and diverse clinical manifestations, and are often misdiagnosed or missed. However, in the early stages of infancy, spasms often occur. When infantile spasms occur, we suggest that genetic testing should be carried out as early as possible, which is helpful for clinical diagnosis and has great value in improving disease prognosis and clinical consultant. The treatment results in our study suggest that timely cessation of spasmodic seizures in infants and young children can improve the patient’s neurodevelopment.

In summary, patients with the pathogenic variants in *IRF2BPL* manifest as infantile spasms during infancy due to epileptic seizures. Most children exhibit developmental delay, some show low muscle tone, and a few others may present nystagmus and microcephaly. Imaging results may suggest brain atrophy. The expression level of *IRF2BPL* varies significantly among different age groups, it increases during the embryonic stage (Bostocyst/embryoid body) and adult stage (Adult), however, its expression level in brain is relatively low compared with other organ tissues which explains the different manifestations in clinical cases. Early infancy is characterized by developmental disorders, but lacking imaging manifestations of severe brain developmental abnormalities. As patient age, they share a highly similar and progressive neurodegeneration process which ultimately develop to severe developmental disorders and gradually developing brain developmental abnormalities, such as brain atrophy.

## Conclusion

In this study, we reported three *de novo* pathogenic variants (two were new, one was reported) of *IRF2BPL* in probands with developmental delay and epilepsy. Our report broadens the genotype of *IRF2BPL*-related developmental disorders and provides animal model phenotypes for future mechanistic research. Then, we for the first time confirmed that disruption of irf2bpl could introduce spontaneous electrographic seizures which mimics key phenotypes in human patients. Importantly, we report the treatment strategy and outcome for our patients who gain some development improvement after their spasms were controlled. Our results suggest that timely cessation of spasmodic seizures can improve the patient’s neurodevelopment.

### Electronic supplementary material

Below is the link to the electronic supplementary material.

Supplementary Material 1

## Data Availability

The data that support the findings of this study are available in the ClinVar database (https://www.ncbi.nlm.nih.gov/clinvar/, accession numbers SCV002771877.1, SCV002771878.1, SCV002771879.1).
