# Supplementary material for: De novo variants of IRF2BPL result in developmental epileptic disorder
Source: Orphanet J Rare Dis. 2024 Mar 13;19:121. doi: 10.1186/s13023-024-03130-z (PMC10938665; doi:10.1186/s13023-024-03130-z)
Supplement: Supplementary file 1 — Supplementary Material 1 [file 13023_2024_3130_MOESM1_ESM.docx]

Author response to the comments:

Dear editor

Thanks for your kind suggestions. The revised file in Manuscript ID: JRD-D-23-00625 entitled “De novo variants of IRF2BPL result in developmental epileptic disorder” have been uploaded again. The manuscript was revised according to the comments of reviewers.

The details of the response to reviewer comments were below:

Reviewer #2: This is a manuscript describing three patients with developmental delay, epilepsy exhibiting as spasms found to have heterozygous variants in IRF2BPL gene. The authors describe the genotype and phenotype of the patients. The authors have demonstrated electrographic epileptiform events seen in irf2bpl crispant zebrafish may correlate to the seizures seen in patients.

These are my comments/suggestions:

1. It would be helpful if the authors can include the phenotype typically seen in IRF2BPL-related neurodevelopmental disorder in the introduction.

Answer: Thank you for the suggestions, and we have added the typically phenotype for IRF2BPL related neurodevelopmental disorder in the introduction.

2. What do you mean by development investigation? Could you elaborate on this?

Answer: Thank you for your question. The “development investigation” here may be not suitable. What we mean is to use some developmental scales, such as “The Gesell scale”, to evaluate the patient's development. We have revised it to “developmental assessment” in the manuscript.

3. What are the 'genetic screens' performed in these patients?

Answer: Thank you for your question. The “genetic screens” here may be not suitable. What we mean is the whole exome sequencing for the patients and their families. We have revised it to “genetic testing” in the manuscript.

4. What are ID and DD?

Answer: Thank you for your suggestions and we regret that we did not provide the full names of these abbreviations. ID is for "Intellectual Disability" and DD is for "Developmental Delay." We have added them in the manuscript.

5. Is there neuroregression seen in patient 1?

Answer: We apologize for the unclear description of this part. Case 1 in our report showed developmental regression. At the age of 3 months, he could raise his head steadily and follow lights and sounds well. After the spasm attack, he experienced developmental regression, manifested as unsteadiness in raising his head, slow reaction to human faces, and inability to find the source of sounds. And we have added it in the manuscript.

6. Could you provide the reference for the statement 'A previous study…atonic seizures' Line 287-290

Answer: Thank you for your suggestions and we have added the reference in the manuscript.

7. What is the exact genotype of the irf2bpl crispants? If it is a IRF2BPL-knock-out zebrafish model, It is not clear to me on how that the findings seen in the zebrafish model conducted by the authors can conclusively correlate to the clinical findings seen in the patients, especially the patients with missense variants in IRF2BPL (patient 1 and 3). The missense variants in these patients may not be truly causing loss of function in IRF2BPL, even though they are predicted to be damaging by multiple prediction algorithm.

Answer: Thank you for the question. We apologize for not providing detailed information in our manuscript. Crispant is a new CRISPR/Cas9 method generates mutant zebrafish. Crispant approach to create knock-out animals by “breaking” a gene in multiple locations (in our report was three). This is a fast and efficient method and is widely used in the construction of disease models.

In response to your concern about the correlation between our zebrafish model findings and clinical observations in patients with missense variants in IRF2BPL, we acknowledge the importance of this issue. Indeed, missense variants may not always lead to a complete loss of function in the gene, as they might retain partial activity or have other effects on protein function.

While our zebrafish model represents a complete loss of IRF2BPL function, it is essential to recognize the limitations in directly extrapolating findings from knockout models to cases with missense variants. To address this limitation, further studies are warranted to investigate the specific effects of missense variants observed in patients, including functional assays to assess their impact on IRF2BPL protein function.

8. The patients who have neuroregression seem to have improved after the spasms/seizure are under control with antiepileptic medication / surgery. Is this reversibility observed in other pediatric patients with infantile spasms/seizures in other publications?

Answer: Thank you for your question. We reviewed previous reports that some patients with epileptic phenotypes use drugs to control, but only the control of epilepsy symptoms was described, and there seemed to be no specific description of the impact on development. Looking at longer-term follow-up, there appear to be no cases where the patient's overall symptoms have improved very well (PMID: 36810721, 37114479). Three years after a patient controlled her tonic-clonic seizures with medication, she still developed myoclonic jerks and unsteady gait (PMID:35525099). Due to the challenges associated with systematic IQ assessment, it was not possible to establish a correlation between the degree of seizure control and later neurologic/cognitive outcomes (PMID: 30166628). The follow-up period of our patients was short, and subsequent patient outcomes are difficult to determine.

9. Which variant has been reported before? Is there any similarities/difference in terms of phenotype of the other patient in comparison to the patient reported in this manuscript?

Answer: Thank you for your question. The variant in case 1 c.1171C>T, p.Arg391Cys was reported before (PMID: 28135719). It was included in large-scale sequencing studies for developmental disorders (McRae *et al.,* 2017). Therefore, other than developmental delay, we do not know the specific clinical phenotype in the reported patient.

10. There is improvement in English language / grammar used in the manuscript. However, there are still a few terminologies /sentence structures used may not be appropriate. Highlighting a few examples as below but will appreciate the authors to review again.

i) Line 386 - 'When infants with spasms' - suggest to rephrase this.

Answer: Thank you for your suggestions and we revised it to “When infantile spasms occur”.

ii)'Spasticity' (line 189) may not be appropriately used in this context. Do you mean 'spasms'?

Answer: Thank you for your reminder and we have revised it to “spasms'”.

iii)I may just refer to as neurodevelopment rather than 'Developmental process'

Answer: Thank you for your suggestions and we revised it to “neurodevelopment”.

iv) What is 'non-genetic' family history?

Answer: Thank you for your query, and we have revised it to “”no family history of genetic disease

v) What does 'low muscular tension' mean? Is it hypotonia?

Answer: Thank you for your suggestions and we revised it to “hypotonia”.
